# Patient-specific three-dimensional evaluation of interface micromotion in two different short stem designs in cementless total hip arthroplasty: a finite element analysis

**DOI:** 10.1186/s13018-022-03329-5

**Published:** 2022-09-29

**Authors:** Arata Kanaizumi, Daisuke Suzuki, Satoshi Nagoya, Atsushi Teramoto, Toshihiko Yamashita

**Affiliations:** 1 Department of Orthopaedic Surgery, School of Medicine, Sapporo Medical University, 291, Minami 1 Jo Nishi 16 Chome, Chuo-ku, Sapporo, Hokkaido 060-8543 Japan; 2 Faculty of Health Science, Hokkaido Chitose College of Rehabilitation, 2-10-10 Satomi, Chitose, Hokkaido 066-0055 Japan; 3 Department of Musculoskeletal Biomechanics and Surgical Development, Sapporo Medical University, Minami 1 Jo Nishi 17 Chome, Chuo-ku, Sapporo, Hokkaido 060-8556 Japan

**Keywords:** Micromotion, Vector, Finite element analysis, Total hip arthroplasty, Cementless short stem

## Abstract

**Background:**

Evaluation of micromotion in various activities in daily life is essential to the assessment of the initial fixation of cementless short stems in total hip arthroplasty. This study sought to evaluate three-dimensionally the micromotion of two types of cementless short stems.

**Methods:**

Two types of stems were used: the Fitmore stem with a rectangular cross-section (rectangular stem) and the octagonal-oval GTS stem with fins (finned stem). Finite element analysis was used to calculate the micromotion of two activities that place a heavy load on the stem (single-leg stance and stair climbing). Three values were measured: the magnitude of micromotion (mean and 95th percentile), the location of micromotion above the 95th percentile value, and the directions of the micromotion vector.

**Results:**

1. There was no significant difference in the magnitude of the micromotion between the rectangular stem and finned stem groups for single-leg stance or stair climbing. 2. In both groups, the micromotion was greatest at the proximal and distal ends. 3. The direction of the micromotion was similar in both groups; internal rotation occurred from the distal to the middle of the stem during stair climbing.

**Conclusions:**

The rectangular stem had comparable initial fixation to that of the finned stem. In both models, the micromotion was greater at the proximal and distal ends. The direction of the micromotion was not dependent on the stem shape but on the direction of the load on the artificial femoral head. These results will be important for stem selection and future stem development.

**Supplementary Information:**

The online version contains supplementary material available at 10.1186/s13018-022-03329-5.

## Introduction

Cementless short stems have been used for hip arthroplasty in an increasing number of cases due to their superiority in femoral bone preservation. Although the medium-term results for cementless short stems are stable, there are some cases in which the stem loosens within a short period due to insufficient initial fixation [1, 2]. To achieve initial fixation, the micromotion of the stem must be suppressed [3, 4]. When the micromotion is less than 30 μm, bone ingrowth occurs between the coated portions of the stem. When the micromotion is more than 150 μm, bone ingrowth is inhibited by connective tissue [4–8]. It is crucial to evaluate the magnitude of micromotion in cementless stems as the stem is subjected to many forces during various daily activities [9, 10]. It is also essential to understand the direction of the micromotion, as subsidence and rotation of the stem may occur in the early postoperative period [11, 12]. There are many unknowns with regard to micromotion for different stem shapes as well as for different activities, and various implant shapes have been devised to achieve stable fixation [1]. The purpose of this study was to evaluate three-dimensionally the micromotion of two types of cementless short stem under loading conditions using the finite element method.

## Materials and methods

**Fig. 1:**
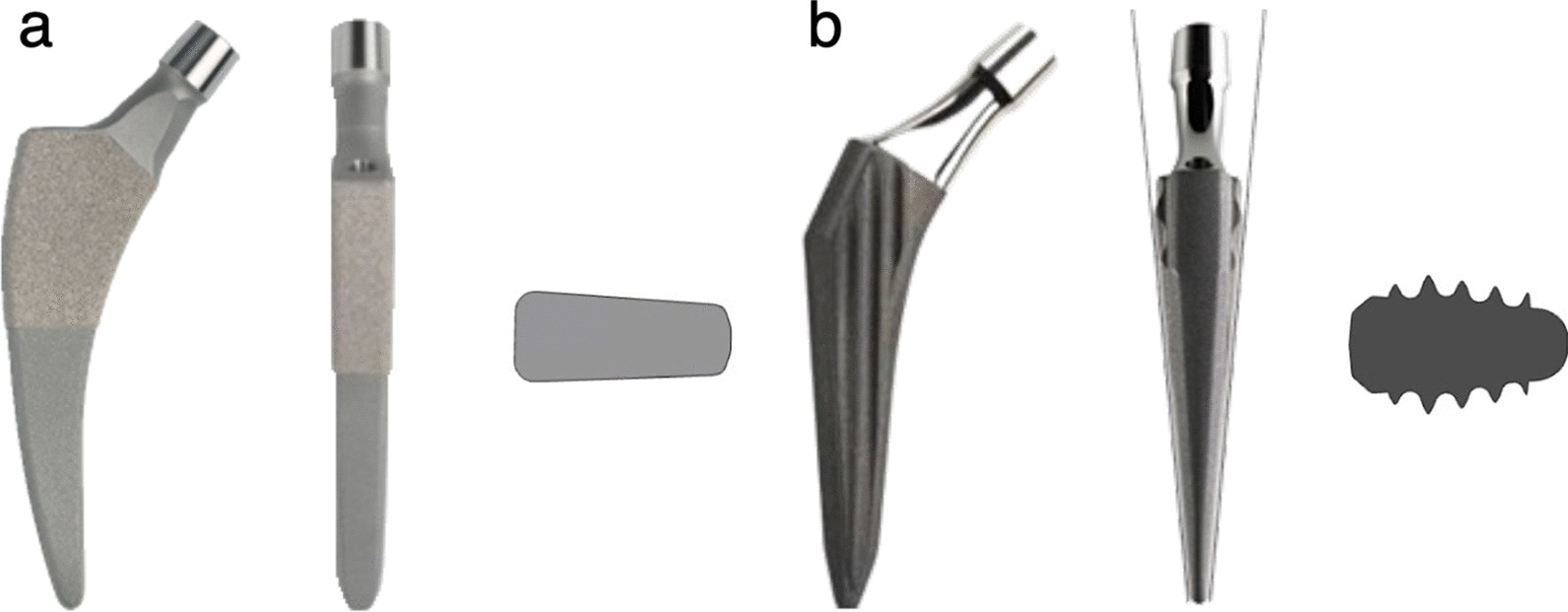
The shapes of the two stems. **a** Anterior view, lateral view, and cross section of the Fitmore stem (rectangular stem). **b** Anterior view, lateral view, and cross section of the GTS stem (finned stem)

The subjects consisted of patients who underwent total hip arthroplasty (THA) with a Fitmore or GTS stem (both stems are manufactured by Zimmer Biomet Holdings, Inc., Indiana, USA) (Fig. 1) at Sapporo Medical University Hospital between April 2016 and March 2018. With regard to the stem used, patients were randomly selected by the permuted block method. All patients underwent computer tomography (CT) imaging for preoperative and postoperative evaluation. Patient selection criteria were as follows: a Dorr classification type B [13] and age less than 75 years. Only femurs with type B Dorr classification were selected to exclude cases with extraordinarily narrow or extensive medullary cavities. Patients with previous femoral osteotomy were excluded.

The Fitmore stem (rectangular stem) is characterized by its rectangular cross section and medial curve, and the GTS stem (finned stem) is characterized by its elliptical octagonal cross section with fins. The surgical approach was antero-lateral (AL), also known as the Orthopädische Chirurgie München (OCM) approach, in all cases. This study was approved by the Institutional Review Board of our hospital (Approval Number: 292-41). CT images (Aquilion PRIME; Toshiba, Tochigi, Japan) were obtained at 120 kV, 70mAs, and a slice thickness of 0.5 mm. CT images were taken from the pelvis to both knee joints before THA and within 2 weeks after THA.

### Finite element analysis

**Fig. 2:**
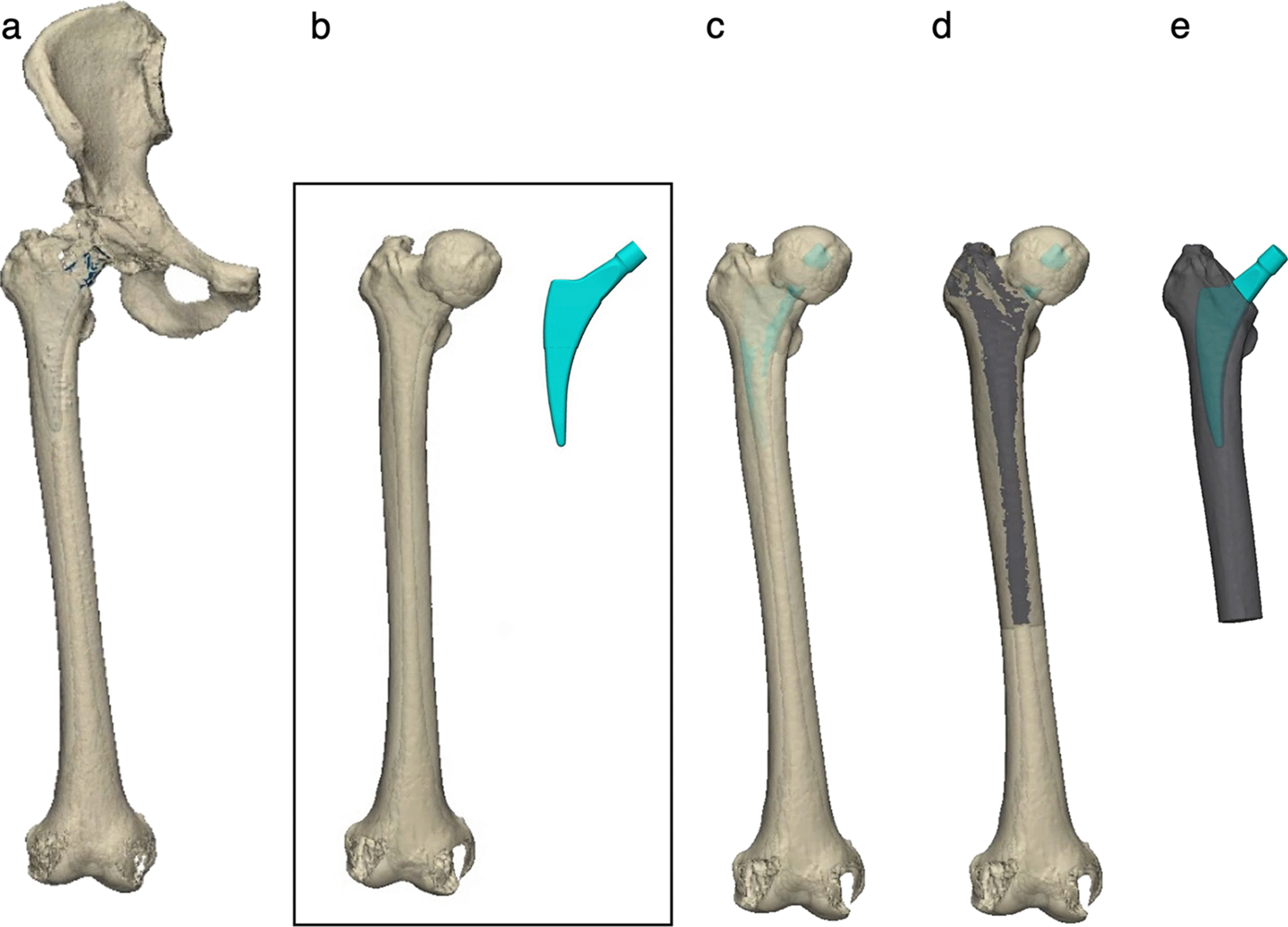
Methods for reproducing postoperative femur-stem models without artifacts. **a** The postoperative femur (including pelvis) and stem with artifacts. **b** Preoperative femur and stem. **c** Superimposed preoperative femur and stem with 3D–3D registration on postoperative femur and stem. **d** Cutting out the femur. **e** Postoperative femur-stem models without artifacts for FEA

As postoperative CT images show strong artifact of the stem, postoperative femur-stem models were created using the following method. First, the pre- and postoperative CT images of all selected patients were converted into standard triangulated language (STL) data using three-dimensional (3D) imaging software (Mimics ver. 23, Materialize, Leuven, Belgium). The femurs and the implanted stems were then three-dimensionally reconstructed from the CT images; values of 200 Hounsfield units (HU) or more were defined as bone [14–16], and those of 2000 HU or more were defined as the stem (Fig. 2a, b). Next, the pre- and postoperative femur models were subjected to 3D–3D registration to reproduce the postoperative femur-stem models without artifacts accurately using 3D modeling software (3-matic ver. 15, Materialize, Leuven, Belgium) (Fig. 2c, d). Finally, the resultant postoperative femur-stem models (Fig. 2e) were read by finite element analysis software (MECHANICAL FINDER ver. 11, Research Center of Computational Mechanics, Japan), which reflected the bone mineral density (BMD) of the femur from the CT values.

Elastic analysis was used for the analysis. Micromotion was defined as the displacement difference at the element nodes of the contact surface between the femur and the stem [17–19]. The finite element analysis models used solid elements (4-node tetrahedron) and shell elements (0.001 mm). The maximum and minimum internal mesh sizes of the femur were 2 mm and 1 mm, respectively, and those of stem were 1 mm and 0.5 mm, respectively. The mean number of elements for the femur and stem was approximately 310,000 and 80,000, respectively, in the rectangular stem group, and 330,000 and 120,000, respectively, in the finned stem group. The average number of elements for the artificial head was 30,000 in both groups. Mesh convergence tests confirmed that the deflection converged at the mesh sizes used in the present study (Additional file 1 Fig. S1). The Young’s modulus of the femur was determined for elastic analysis as follows. The CT value (HU) of each mesh was converted to BMD (*ρ* (g/cm^3^)) using the standard calibration curve of the finite element analysis software, as in previous reports [20, 21], and the Young’s modulus (*E* (MPa)) was calculated from the BMD of each mesh based on the prediction equation of Morgan et al. [17, 22] (Table 1). The Young's modulus of the stem and artificial head was 109 GPa. The Poisson’s ratio of the stem and artificial head was 0.28 and that of the femur was 0.40 [20, 23].

**Table 1:** Parameters for finite element analysis

	Femur	Rectangular stem	Finned stem	Artificial head
Young's modulus (MPa)	10 + 6850ρ^1.49^†	109 × 10^3^	109 × 10^3^	109 × 10^3^
Poisson’s ratio	0.4	0.28	0.28	0.28
Friction coefficient	–	0.64, 0.49*	0.49	–

†*ρ*(g/cm^3^) = (0.945 HU + 1.35) × 1.0^−3^ (HU > − 1), *ρ*(g/cm^3^) = 0 (HU ≦ − 1)

*ρ*: Bone mineral density, HU: CT value

*The proximal and distal coated portions of the rectangular stem, respectively

**Fig. 3:**
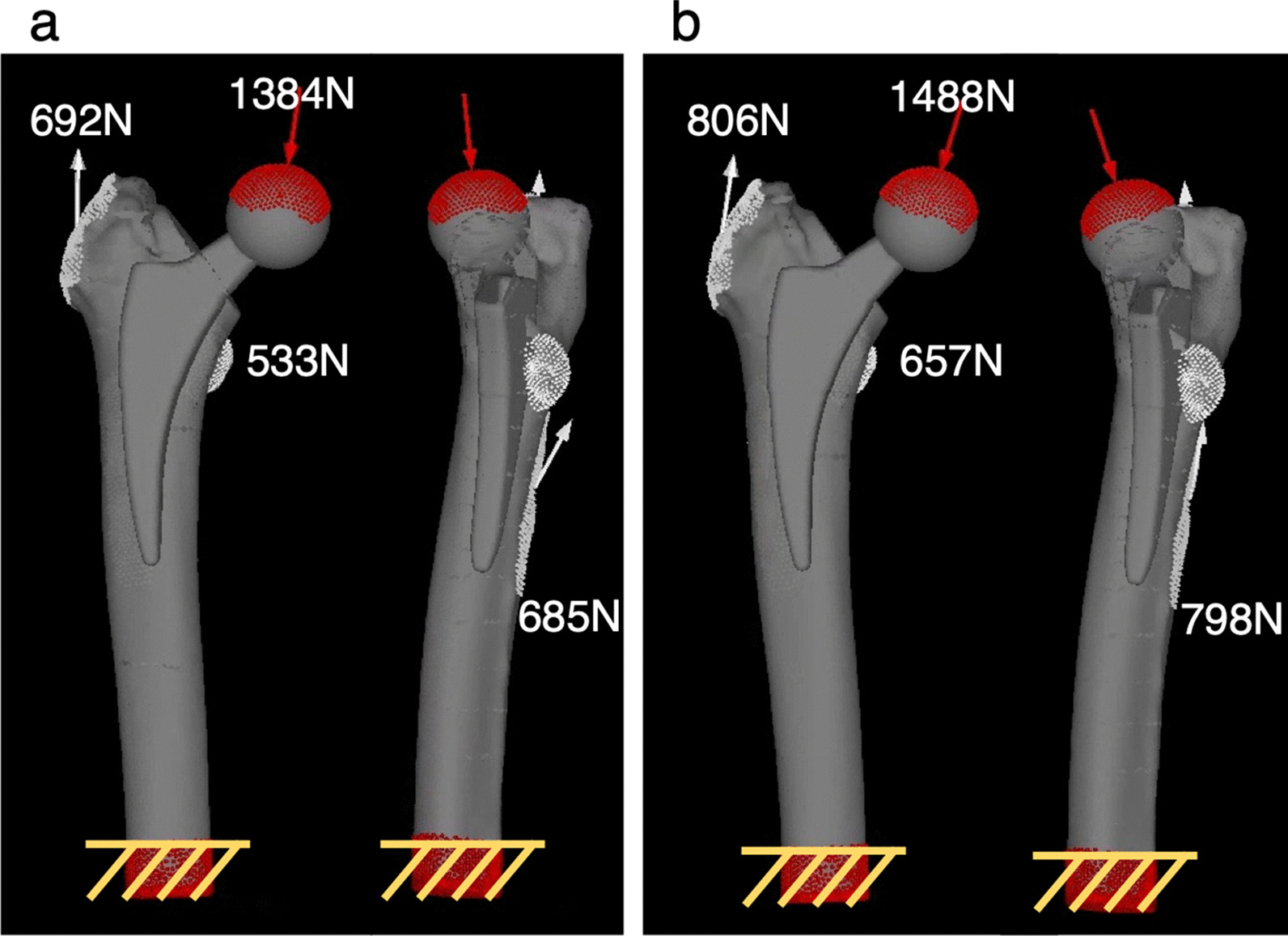
Boundary conditions for the finite element method in two different activities for a body weight of 50 kg. **a** Single-leg stance. **b** Stair climbing. Red arrows and areas indicate the direction and area of load on the artificial head, respectively. The white arrows and area indicate the direction and area of tension in the greater trochanter, lesser trochanter, and gluteal tuberosity. The yellow shaded areas are fixed in all directions

The maximum load values for two activities, “single-leg stance” and “climbing stairs,” were used for the load conditions. As the rotation torque is small for single-leg stance and large for stair climbing [24], it is possible to evaluate both vertical and rotational directions. The load was calculated in relation to the patient's weight. For example, when a patient weighing 50 kg stood on one leg, a load of 1384 N was applied to the prosthetic head at 16° in the anterior forehead plane and 4° in the sagittal plane. Tensile forces of 692 N, 533 N, and 685 N were applied to the greater trochanter, lesser trochanter, and gluteal tuberosity, respectively [24–26] (Fig. 3a). For stair climbing, a load of 1488 N was applied to the artificial bone head at 20° in the frontal plane and 18° in the sagittal plane, and tensile forces of 806 N, 657 N, and 798 N were applied [24, 26, 27] (Fig. 3b). The distal end of the femur was fully constrained. The friction coefficients of the contact surfaces between the femur and the stem were as follows: 0.64 and 0.49 for the proximal and distal coated portions of the rectangular stem, respectively, and 0.49 for the coated portion of the finned stem [28].

### Data analysis

**Fig. 4:**
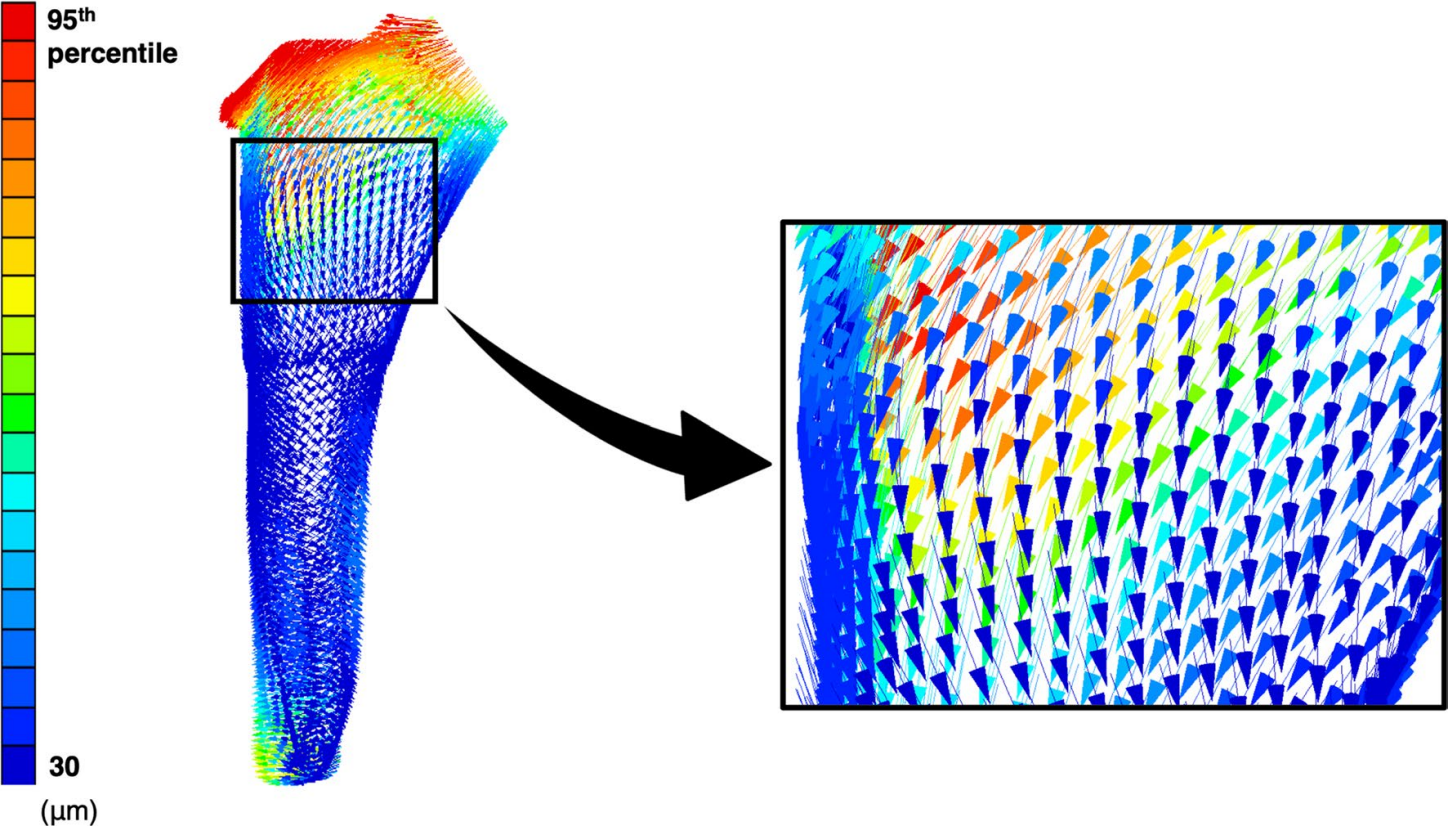
Vectoral representation of the micromotion. The vectors are multiplied by 150. The black arrow indicates a magnified view

Outcome measures were the value (mean and 95th percentile) of the micromotion of the stem calculated by finite element analysis, the area in which micromotion with a value greater than the 95th percentile was distributed, and the direction of the micromotion vector. The 95th percentile value was used to minimize the influence of outliers, as the maximum value could be affected by outliers. The vectors were displayed as vectors from the starting point of each node, and the length of the vectors was multiplied by 150 and illustrated using graphing software (Graph-R for Mac ver. 1.24.3, S-NEXT, Iwate, Japan) (Fig. 4). In addition, micromotion below 30 µm was judged to represent good initial fixation [4–8] and the lowest value of micromotion was shown as 30 µm. All statistical analyses were performed using statistical software (Stat Plus: mac Pro ver. 7, AnalystSoft Inc., VA, USA) and Microsoft Excel (for Mac ver. 16, Microsoft, USA). Statistics were performed using paired t-test and Mann–Whitney *U* test at a significance level of *p* = 0.05. All data are presented as mean ± standard deviation.

## Results

Twenty patients (3 males and 17 females) with a mean age at surgery of 62.4 ± 7.9 years and a mean body mass index (BMI) of 24.7 ± 3.0 were included. Ten patients had rectangular stems and 10 had finned stems (Table 2). No stem subsidence was observed on radiographs up to 1 year postoperatively.

**Table 2:** Patient demographics

	Rectangular stem	Finned stem	Total	*p*-value
Number of joints	10	10	20	
Age (years)	59.1 ± 7.4	61.1 ± 7.3	60.1 ± 7.4	0.569
Sex, *n* (%)				0.556
Male	2 (20%)	1 (10%)	3 (15%)	
Female	8 (80%)	9 (90%)	17 (85%)	
Height (cm)	161.1 ± 7.0	154.8 ± 3.9	158.0 ± 6.5	0.033
Body weight (kg)	63.7 ± 8.3	59.5 ± 8.8	61.6 ± 8.8	0.313
BMI (kg/m^2^)	24.5 ± 2.6	24.8 ± 3.4	24.7 ± 3.0	0.837
Cause of THA, *n* (%)				
OA (due to dysplasia)	9 (90%)	9 (90%)	18 (90%)	1.000
ONFH	1 (10%)	1 (10%)	2 (10%)	
Canal flare index	3.69 ± 0.39	3.71 ± 0.37	3.53 ± 0.45	0.133

**Fig. 5:**
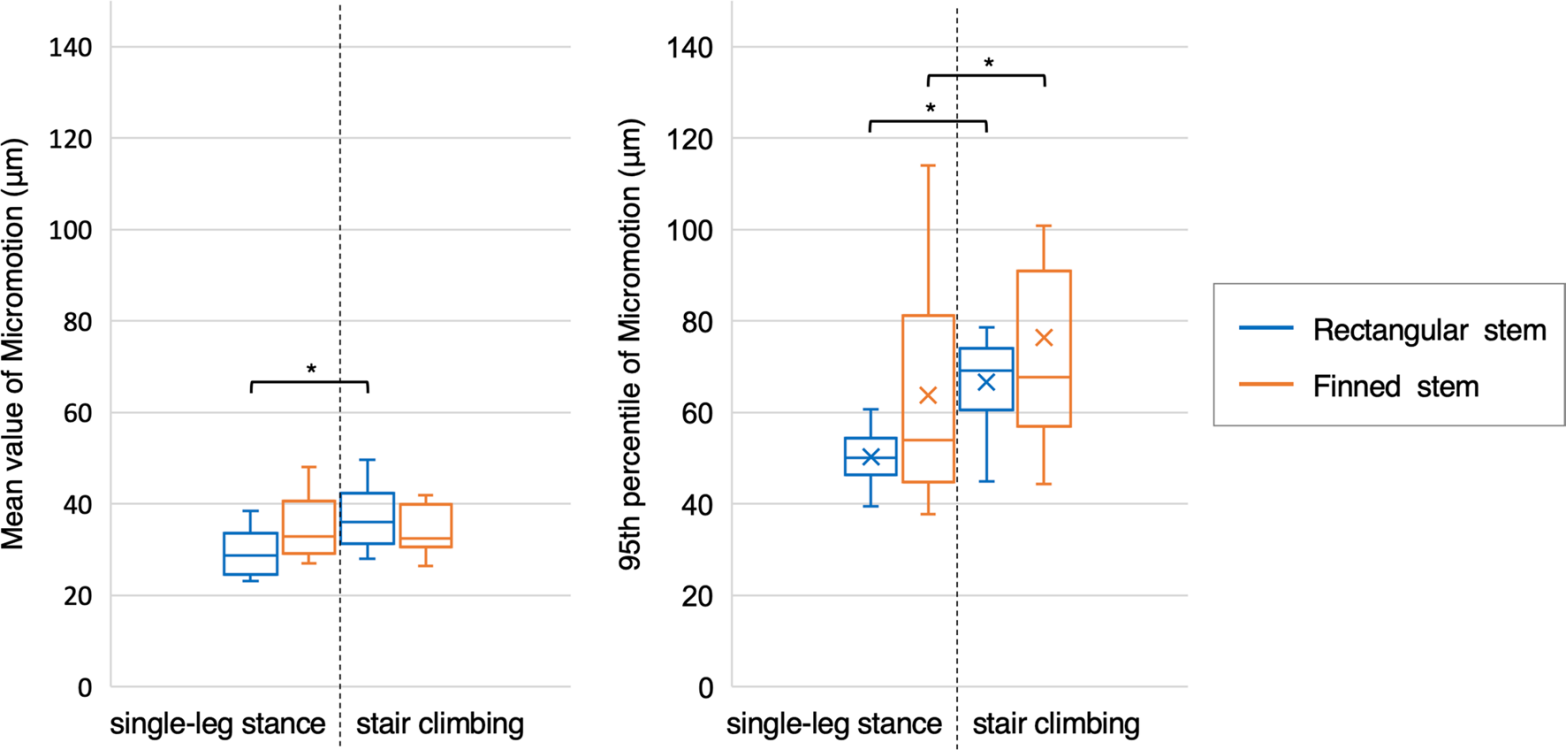
Boxplot of mean values and 95th percentiles of the micromotion for each of the 10 cases in the rectangular stem and finned stem groups during single-leg stance and stair climbing

In the rectangular stem group, the mean value of the micromotion was 29.9 ± 5.0 μm for single-leg stance and 37.4 ± 7.2 μm for stair climbing, with that for stair climbing being significantly larger (*p* < 0.01) (Fig. 5). In the finned stem group, the mean value of the micromotion was 34.8 ± 6.6 μm for single-leg stance and 35.5 ± 7.6 μm for stair climbing, and there was no significant difference between the two activities. Further, there were no significant differences in the mean value of the micromotion between the two groups. The 95th percentile values of the micromotion were 50.3 ± 5.9 μm for single-leg stance and 66.6 ± 10.4 μm for stair climbing in the rectangular stem group, and 63.8 ± 23.3 μm and 76.4 ± 28.3 μm, respectively, in the finned stem group, with the values for stair climbing significantly larger in both groups (*p* < 0.01). There were no significant differences in the 95th percentile values of the micromotion between the two groups.

**Fig. 6:**
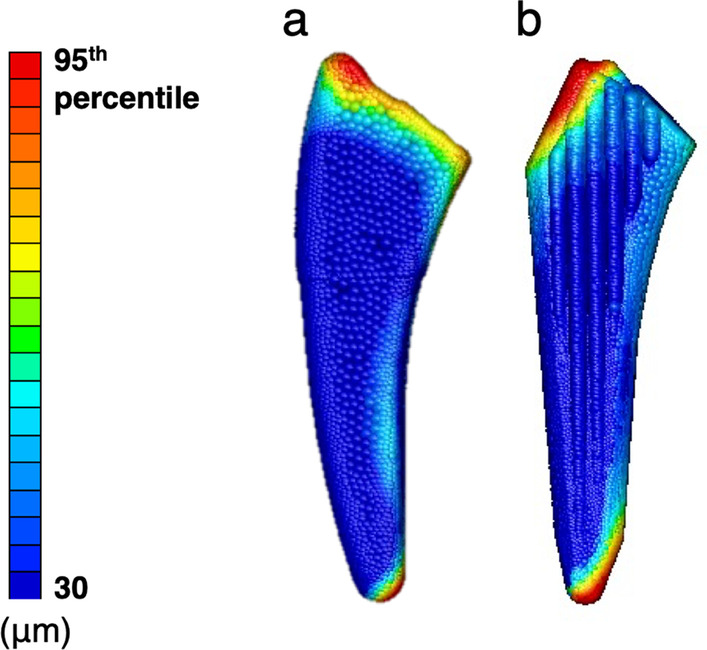
Distribution of micromotion. **a** Rectangular stem. **b** Finned stem

In both groups, micromotion above the 95th percentile value was found at the proximal and distal ends during single-leg stance and stair climbing (Fig. 6). Micromotion above the 95th percentile value was found at the proximal end in all patients during single-leg stance and stair climbing, whereas micromotion above the 95th percentile value at the distal stem end was observed in eight patients in the rectangular stem group and two patients in the finned stem group during single-leg stance, and in five patients in the rectangular stem group and five patients in the finned stem group during stair climbing.

**Fig. 7:**
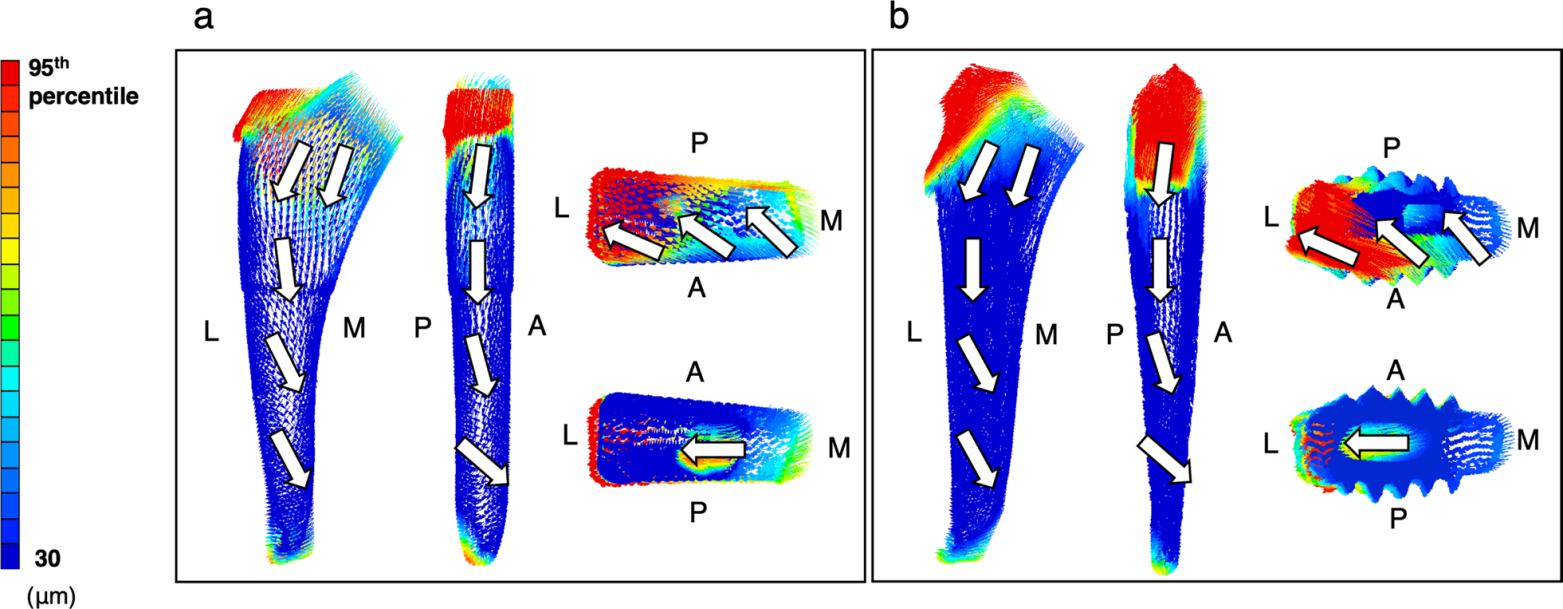
Micromotion vectors of stems during single-leg stance. Anterior, posterior, superior, and inferior views. **a** Rectangular stem. **b** Finned stem. The white arrow indicates the direction of the vector. A, anterior; L, lateral; M, medial; P, posterior

**Fig. 8:**
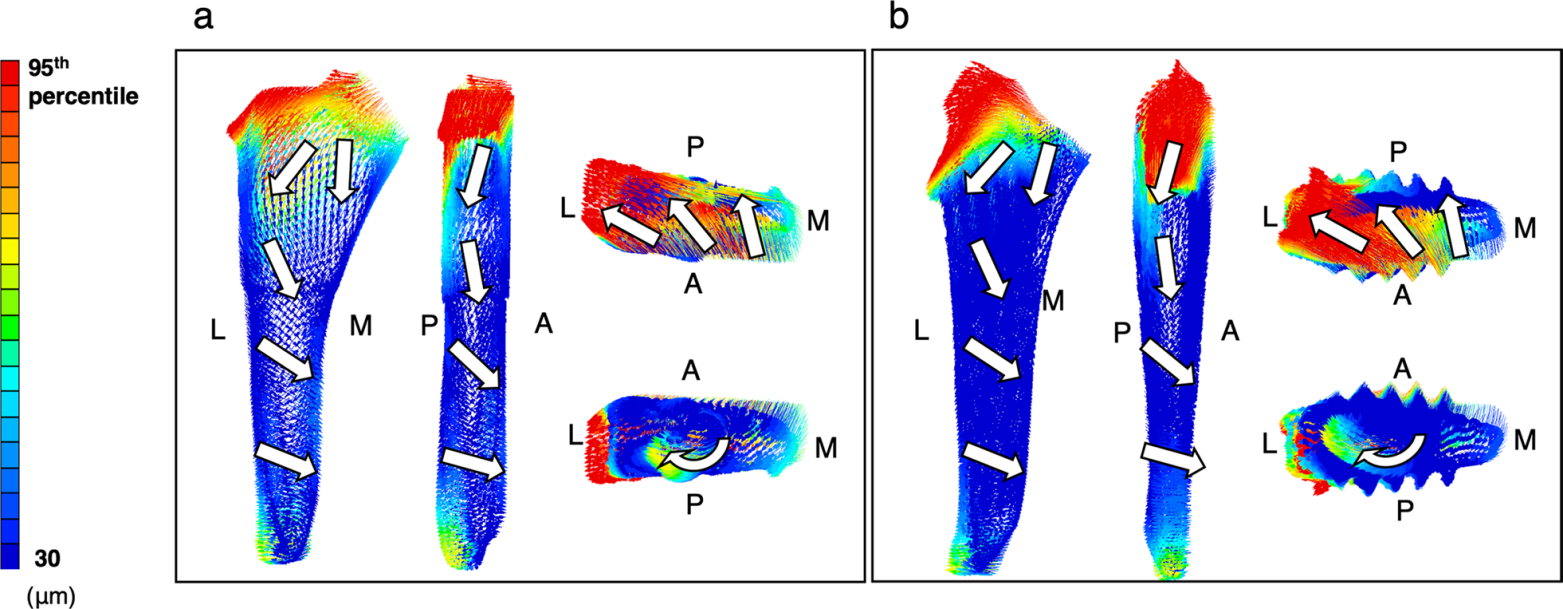
Micromotion vectors of stems during stair climbing. Anterior, posterior, superior, and inferior views. **a** Rectangular stem. **b** Finned stem. The white arrow indicates the direction of the vector. A, anterior; L, lateral; M, medial; P, posterior

The micromotion vectors were similar for the same activity in the two groups (Figs. 7 and 8). The directions of the micromotion vectors of stair climbing were more internally rotated than those of single-leg stance.

## Discussion

This study is the first to evaluate the direction of the micromotion of the stem in three dimensions. To evaluate the initial fixation of the cementless short stems, we need to evaluate micromotion under load [9, 10, 12]. The evaluation of micromotion in cementless stems has previously focused on the magnitude and two-dimensional direction (anterior/posterior/medial/lateral); however, it is also essential to evaluate the three-dimensional direction of micromotion. In this study, we evaluated both the three-dimensional direction and magnitude. The mean and 95th percentile values for the magnitude of the micromotion of the rectangular stem and the octagonal-oval cross-sectioned finned stem did not significantly differ for either single-leg stance or stair climbing. For both stems, the micromotion above the 95th percentile occurred at the proximal and distal ends of the stem. Furthermore, the vector-based assessment showed that stair climbing produced larger anterior-medial-posterior rotational (internal rotational) micromotion in the distal part of the stem than did single-leg stance.

For the initial fixation of cementless stems, it is important that they have excellent rotational stability [29]. However, few reports have evaluated rotational stability. In activities in daily life, the rotational torque applied to the stem is low during single-leg stance and high during stair climbing [24]. In the present study, we also found rotational micromotion during stair climbing. In addition, the mean and 95th percentile values of micromotion within both stem groups were significantly larger during stair climbing than during single-leg stance for most comparisons. This cause may be due to the increased load on the artificial head during stair climbing compared to during single-leg stance and the increased internal rotation torque around the stem axis. On the other hand, the mean and 95th percentile values of micromotion in the two stem groups did not differ significantly for single-leg stance or stair climbing. These results suggest that the rectangular stem with no fins has comparable initial fixation to the octagonal-oval finned stem.

Al-Dirini et al. [17] reported that micromotion above the 95th percentile values occurred at the proximal and distal ends in standard types of stems with and without collars. In the present study, rectangular stems and finned stems both produced micromotion above the 95th percentile values at the proximal and distal ends. In particular, micromotion above the 95th percentile values was observed at the proximal end in all cases. These results suggest that micromotion is larger at the proximal and distal ends in short stems as well as in standard stems. Therefore, it is necessary to suppress the micromotion at the proximal and distal ends to reduce the maximum micromotion. However, to suppress the micromotion at the proximal and distal ends, it is necessary to devise a coating position for the stem and improve the implanted direction of the stem in the future.

No reports to date have evaluated the direction of micromotion three-dimensionally during multiple activities. In the present study, the direction of micromotion was assessed in three dimensions using three-dimensional vectors. In this study, it was found that micromotion with internal rotation from the middle to the distal part of the stem, regardless of the stem shape, occurred more during stair climbing than during single-leg stance. This result suggests that the direction of micromotion in a cementless short stem is not dependent on the shape of the stem but on the direction of loading on the bone head by the activity. By evaluating the micromotion using three-dimensional vectors, it became possible to evaluate the behavior of the stem during early postoperative activities.

One limitation of this study is that the number of cases was small: ten rectangular stems and ten finned stems. However, the strength of this study is that it compares not only a single activity but two different activities with different degrees of rotational torque in daily life. In addition, this study only uses finite element analysis and no validation based on cadaver experiments. However, the same method was used in previous reports where validation was obtained by finite element analysis and cadaver experiments [18, 19]. Furthermore, the same method was used in previous reports on the finite element analysis of micromotion alone [17]. Unlike conventional finite element analysis in which the stem implanted position is simulated, the postoperative stem placement position for each patient in this study is reproduced by 3D–3D registration using CT images taken before and after surgery. Therefore, the finite element analysis based on the accurate reproduction of the position of the surgically implanted stem for each patient is considered to represent the magnitude and direction of the micromotion under actual clinical conditions. Thirdly, each physiological load was assessed one time. The CT images used in this study were obtained within 2 weeks after THA; that is, the CT images in this study were taken after the patient had already started walking. Therefore, the positions of the stems in this study were obtained after at least several hundred cycles of physiological loading, and the effect of initial subsidence on micromotion was considered almost negligible. Finally, as some cases did not use a phantom for evaluating bone mineral density, the standard calibration curve of the finite element analysis software was used for the calculation, as in previous reports [20, 21].

## Conclusions

Using a patient-specific postoperative femoral model, we evaluated the three-dimensional micromotion as obtained from finite element analysis of two types of cementless short stem. The rectangular stem without fins had a comparable initial fixation to the octagonal-oval finned stem. In both models, micromotion was greatest at the proximal and distal ends. Furthermore, the direction of the micromotion was similar for the same activity in both models, suggesting that the direction of the micromotion may depend on the type of motion regardless of the stem shape. By using the three-dimensional vector evaluation of micromotion, we could analyze not only the magnitude but also the three-dimensional direction of the micromotion. The three-dimensional vector evaluation of micromotion in this study is thought to be helpful for stem selection and stem development.

## Supplementary Information

**Additional file1**. **Figure S1**: Mesh convergence Analysis of maximum deflection and number of elements. The largest number of elements is in this study.

## Data Availability

The datasets generated and analyzed during the current study are available from the corresponding author on reasonable request.

## Abbreviations

THA: Total hip arthroplasty
CT: Computed tomography
HU: Hounsfield units
BMD: Bone mineral density
BMI: Body mass index
OA: Osteoarthritis
ONFH: Osteonecrosis of the femoral head
3D: Three-dimensional
