# Supplementary figures and images for: Patient-specific three-dimensional evaluation of interface micromotion in two different short stem designs in cementless total hip arthroplasty: a finite element analysis
Source: J Orthop Surg Res. 2022 Sep 29;17:437. doi: 10.1186/s13018-022-03329-5 (PMC9524017; doi:10.1186/s13018-022-03329-5)

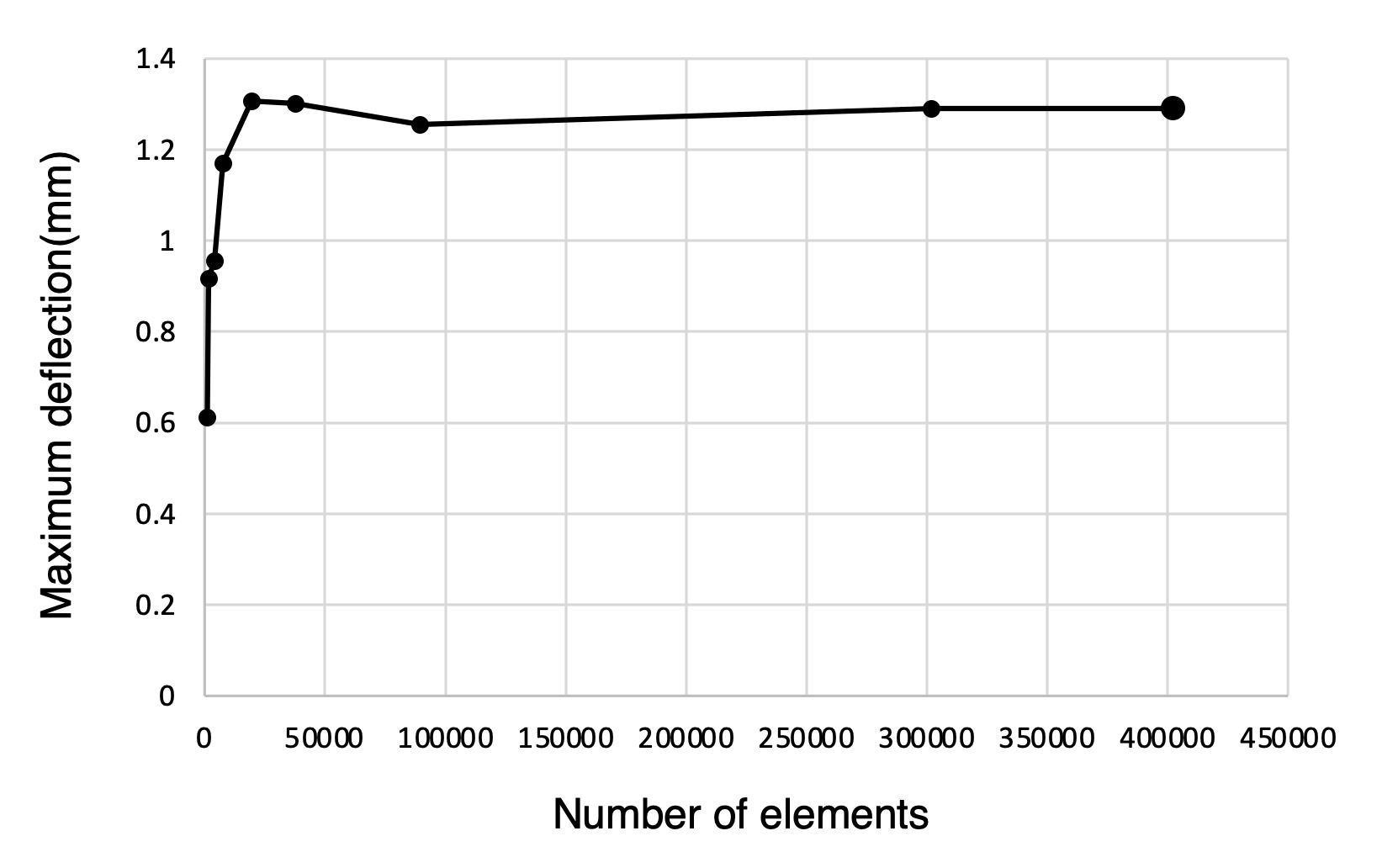

Supplement: Supplementary file 1 — Additional file1. Figure S1: Mesh convergence Analysis of maximum deflection and number of elements. The largest number of elements is in this study. [file 13018_2022_3329_MOESM1_ESM.tif]
